# Eurycomanone and Eurycomanol from *Eurycoma longifolia* Jack as Regulators of Signaling Pathways Involved in Proliferation, Cell Death and Inflammation

**DOI:** 10.3390/molecules190914649

**Published:** 2014-09-16

**Authors:** Shéhérazade Hajjouli, Sébastien Chateauvieux, Marie-Hélène Teiten, Barbora Orlikova, Marc Schumacher, Mario Dicato, Chee-Yan Choo, Marc Diederich

**Affiliations:** 1 Laboratoire de Biologie Moléculaire et Cellulaire du Cancer (LBMCC), Hôpital Kirchberg, 9, Rue Edward Steichen, Luxembourg L-2540, Luxembourg; E-Mails: sheherazade.hajjouli@lbmcc.lu (S.H.); marie_helene.teiten@lbmcc.lu (M.-H.T.); marc.schumacher@lbmcc.lu (M.S.); mdicato@gmail.com (M.D.); 2 Department of Pharmacy, College of Pharmacy, Seoul National University, Building 20 Room 303, 1 Gwanak-ro, Gwanak-gu, Seoul 151-742, Korea; E-Mails: s.chateauvieux@snu.ac.kr (S.C.); barboraorlikova@snu.ac.kr (B.O.); 3 MedChem Herbal Research Group, Faculty of Pharmacy, Universiti Teknologi MARA, Puncak Alam, Selangor 42300, Malaysia; E-Mail: choo715@puncakalam.uitm.edu.my; 4 Drug Discovery & Health Core, Universiti Teknologi MARA, Shah Alam, Selangor 40450, Malaysia

**Keywords:** eurycomanone, eurycomanol, *Eurycoma longifolia* Jack, NF-κB, proliferation, cell death

## Abstract

Eurycomanone and eurycomanol are two quassinoids from the roots of *Eurycoma longifolia* Jack. The aim of this study was to assess the bioactivity of these compounds in Jurkat and K562 human leukemia cell models compared to peripheral blood mononuclear cells from healthy donors. Both eurycomanone and eurycomanol inhibited Jurkat and K562 cell viability and proliferation without affecting healthy cells. Interestingly, eurycomanone inhibited NF-κB signaling through inhibition of IκBα phosphorylation and upstream mitogen activated protein kinase (MAPK) signaling, but not eurycomanol. In conclusion, both quassinoids present differential toxicity towards leukemia cells, and the presence of the α,β-unsaturated ketone in eurycomanone could be prerequisite for the NF-κB inhibition.

## 1. Introduction

Historically, nature has been the major source of bioactive compounds used in both traditional and modern medicine. During the last 30 years, 1,130 drugs have been developed directly from bioactive natural compounds, and altogether, plants are of great interest for pharmacological studies and for the development of new drugs. Currently about 128 anticancer drugs are of natural origins [1].

**Figure 1:**
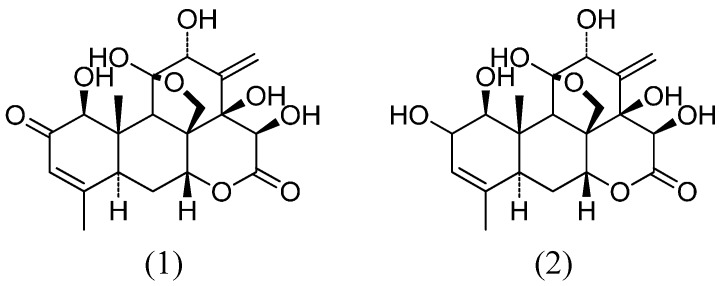
Quassinoid structures of eurycomanone (1) and eurycomanol (2).

*E. longifolia* is studied in Malaysia, where many pharmaceutical preparations are freely available. *E. longifolia* is rich in quassinoids, triterpenes, squalene derivatives, biphenylneolignans, canthin-6-ones and β-carboline alkaloids. The bitter taste of the plant is contributed by the quassinoids. The majority of these components were found in the roots, witnessing the richness of secondary metabolites from this medicinal plant [2]. The major quassinoid, eurycomanone, and its derivative, eurycomanol (Figure 1), were found in most of the collected root samples. Compounds from the bark, leaves and fruits are also known for their cytotoxic effects and present antimalarial, aphrodisiac, anti-anxiety and anti-ulcer potential.

Physiological activation of nuclear factor (NF)-κB is necessary for cell survival [3]; however, its deregulated expression characterizes cancer, inflammation or autoimmune diseases. Thus, abnormal regulation of this transcription factor is found in many cancer types, including chronic lymphocytic leukemia and lymphoid B-cell lymphomas. Currently, NF-κB signaling is considered as an interesting target for anticancer drug development [4,5,6,7]. In addition, NF-κB also contributes to tumor development by activating anti-apoptotic genes, eventually leading to resistance against chemo- and radio-therapy.

Recent research showed that many fruits and vegetables contain molecules with chemopreventive and anti-cancer properties, especially by inhibiting key cell signaling pathways, including signal transducer and activator of transcription (STAT), int/Wingless (WNT) and NF-κB. Active molecules described to inhibit this pathway include chalcones [8,9], curcumin [10], goniothalamin [11,12], quassinoids [13] or cardenolides [14]. In addition, natural marine compounds, such as heteronemin isolated from *Porifera hyrtios* [15], act as potent anti-proliferative and cytotoxic natural compounds with anti-NF-κB potential [6,16,17,18]. Finally, some fungi synthesize valuable compounds, such as clinically used doxorubicin, embellicines [19] or altersolanol [20].

As recent reports underlined the importance of selective cytotoxicity towards cancer cells, we describe here anti-inflammatory and anti-leukemic activities of two compounds from *E. longifolia* with differential NF-κB inhibition potential and differing by a α,β-unsaturated ketone in their structures.

## 2. Results and Discussion

### 2.1. Results

#### 2.1.1. Eurycomanone and Eurycomanol Specifically Affect Cancer Cell Viability and Proliferation

**Figure 2:**
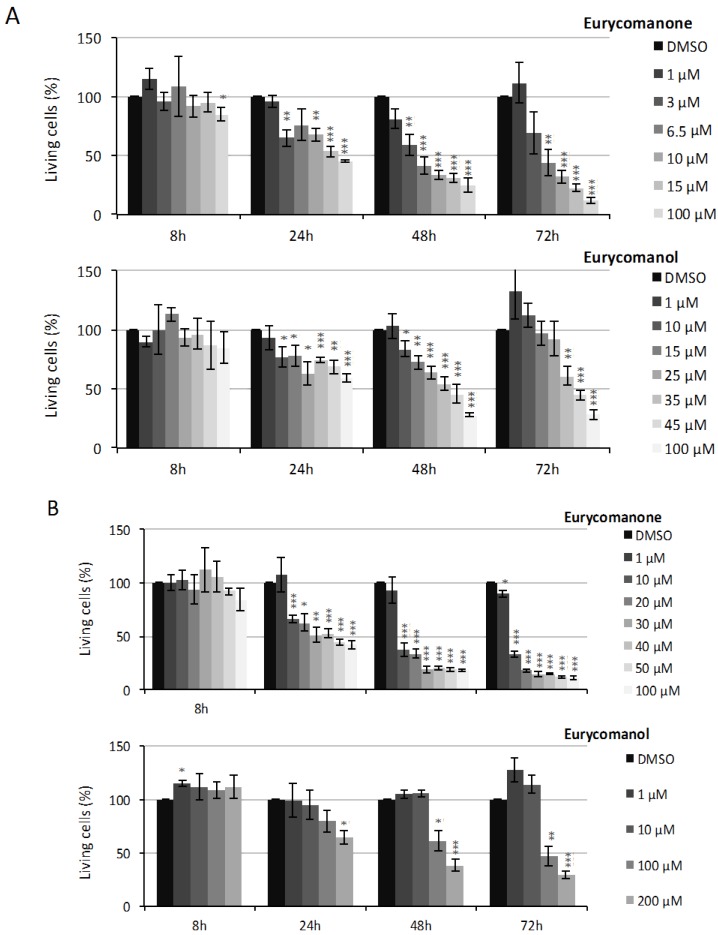
The effect of eurycomanone and eurycomanol on K562 (**A**) and Jurkat (**B**) cell viability. Cell viability was assessed by the trypan blue exclusion test. DMSO corresponds to solvent-treated control. Each value is the mean ± SEM of three independent experiments. *, ** or *** indicate a significant difference compared to the negative control analyzed by the *t*-test (* *p* ˂ 0.05, ** *p* < 0.01, *** *p* < 0.001).

Our results show that eurycomanone and eurycomanol decrease leukemia cell viability dose- and time-dependently (Figure 1). The IC_50_ values at 8, 24, 48 and 72 h are detailed in Table 1. At 72 h, the IC_50_ values are 5.7 and 46.4 µM for K562 (Figure 2A) and of 6.2 and 90.7 µM for Jurkat cells (Figure 2B), for eurycomanone and eurycomanol, respectively.

**Table 1:** The effect of eurycomanone and eurycomanol on K562 and Jurkat cell viability. IC_50_ values were determined using Prism 6.0 (GraphPad), based on cell quantification performed with the trypan blue exclusion test.

Cells	Compound	8 h	24 h	48 h	72 h
K562	Eurycomanone	>150 µM	48.92 µM	4.25 µM	5.7 µM
	Eurycomanol	>150 µM	>150 µM	39.78 µM	46.4 µM
Jurkat	Eurycomanone	>150 µM	40.2 µM	5.7 µM	6.2 µM
	Eurycomanol	>150 µM	>150 µM	118.6 µM	90.7 µM

**Figure 3:**
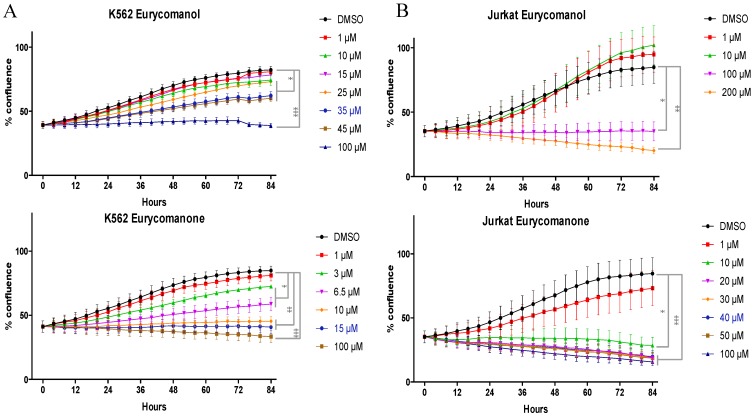
The effect of eurycomanone and eurycomanol on K562 (**A**) and Jurkat (**B**) cell proliferation. Cell proliferation was evaluated by using the IncuCyte^TM^ Life-Cell Imaging System. DMSO corresponds to solvent-treated control. Each value is the mean ± SEM of three independent experiments. The asterisk indicates a significant difference compared to the negative control analyzed by the *t*-test (* *p* < 0.05, ** *p* < 0.01, *** *p* < 0.001).

As reduced viability could be due to both reduced proliferation and/or increased cell death rates, we investigated the effect of eurycomanone and eurycomanol on leukemia cell proliferation rates during 84 h by using an IncuCyte^TM^ video microscopy-based approach (corresponding video clips are provided as Supplementary Materials). Our results confirmed that both compounds induce time- and dose-dependent cytostatic effects (Figure 3). IC_50_ values at 8, 24, 48 and 72 h are detailed in Table 2.

**Table 2:** The effect of eurycomanone and eurycomanol on K562 and Jurkat cell proliferation. IC_50_ values were determined using Prism 6.0 (GraphPad), based on leukemia cell proliferation rates during 84 h by using video microscopy.

Cells	Compound	8 h	24 h	48 h	72 h
K562	Eurycomanone	>150 µM	>150 µM	>150 µM	14.2 µM
	Eurycomanol	>200 µM	>200 µM	>200 µM	143.8 µM
Jurkat	Eurycomanone	>150 µM	>150 µM	9.58 µM	5.89 µM
	Eurycomanol	>150 µM	>150 µM	102.1 µM	93.9 µM

**Figure 4:**
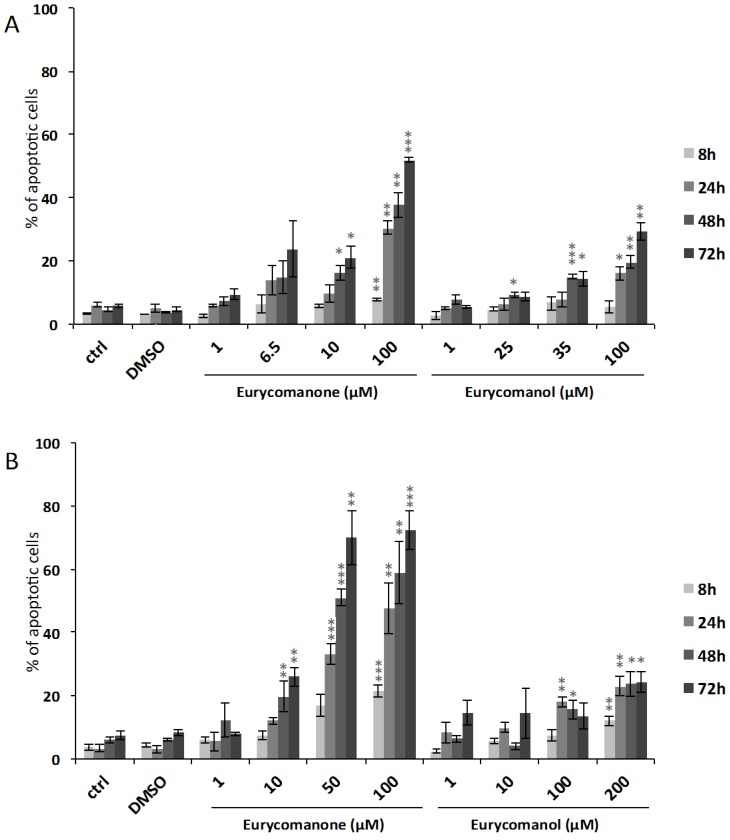
The effect of eurycomanone and eurycomanol on the induction of apoptosis in K562 (**A**) and Jurkat (**B**) cells. Apoptosis was evaluated by quantification of cells presenting fragmented nuclei by fluorescence microscopy after Hoechst staining. DMSO corresponds to solvent-treated control. Each value is the mean ± SEM of three independent experiments. * indicates a significant difference compared to the negative control analyzed by the *t*-test (* *p* < 0.05, ** *p* < 0.01, *** *p* < 0.001).

In addition to reduced proliferation rates, both compounds also induced a cytotoxic reaction. Accordingly, quantification of cells with condensed nuclei after Hoechst staining at the same doses and conditions (Figure 4) show a dose- and time-dependent increase of apoptotic cells reaching 52% and 29% for K562 cells treated during 72 h with 100 µM eurycomanone and 100 µM eurycomanol, respectively. Jurkat cells appeared to be more sensitive to eurycomanone, as we quantified 72% of apoptotic cells after 72 h of treatment with 100 µM.

Here, we compare the results obtained with the quassinoids from *E. longifolia* with previously tested compounds. Our present results show that the inhibition of cell viability induced by eurycomanone remains modest and IC_50_ values are higher compared to most compounds tested under the same conditions in K562 cells (Table 3). Interestingly, eurycomanone inhibits NF-κB activity in a low micromolar range after eight hours of treatment in the absence of any toxicity (Table 3 and Section 2.1.3).

**Table 3:** Comparison of the effect of eurycomanol and eurycomanone on inhibition of viability, proliferation and NF-κB activity. IC_50_ values were measured in K562 cells after the indicated treatment times.

Compound	Inhibition of Cell Viability at 24 h (µM)	Inhibition of Cell Proliferation at 72 h (µM)	Inhibition of NF-κB Activity at 8 h (µM)	Reference
Eurycomanol	>150	143.8	35.6	present study
Eurycomanone	48.92	14.2	6.6	present study
4-Hydroxychalcone	31	nd	24	[9]
4-Methoxychalcone	35	nd	29	[9]
Altersolanol A	4	1	0.8	[20]
Butein	13	nd	38	[9]
Embellicine A	2.9	nd	3	[19]
Embellicine B	0.3	0.5	0.4	[19]
Flavokawain C	13	nd	8	[9]
Goniothalamin	>20	nd	7	[12]
Homobutein	29	nd	38	[9]
Isoliquiritigenin	44	nd	32	[9]
Phloretin	59	nd	41	[9]
Tetraacetylaltersolanol	2.5	nd	4.6	[20]

**Figure 5:**
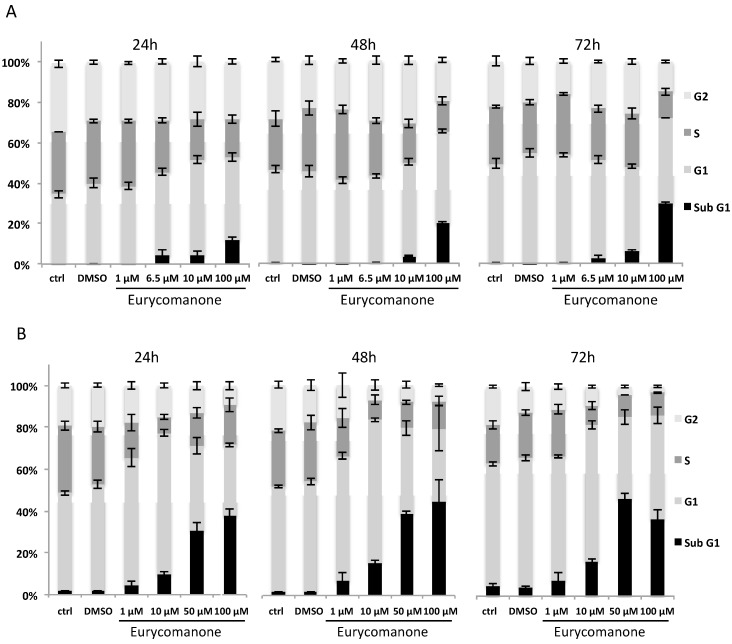
The effect of eurycomanone on the cell cycle distribution of K562 (**A**) and Jurkat (**B**) cells. Cell cycle analysis of K562 and Jurkat cells treated with different concentrations of eurycomanone during 24, 48 and 72 h was performed by flow cytometry. DMSO corresponds to solvent-treated control. Each value is the mean ± SD of three independent experiments.

The results were further confirmed by investigating the effect of eurycomanone and eurycomanol on K562 and Jurkat cell cycle distribution by flow cytometry. A time- and dose-dependent increase of apoptotic (subG1) cells was observed. Jurkat cells were particularly sensitive to eurycomanone treatment with 38% of dead cells at 24 h with 100 µM. K562 cells exhibited 12% cell death under the same conditions (Figure 5). The percentage of cell death by apoptosis (Figure 5) was less than the impact observed on cell viability (Figure 2). This discrepancy is most likely due to the fact that cells are rapidly damaged and their nuclei disintegrated; therefore, they can no longer be observed by Hoechst staining.

**Figure 6:**
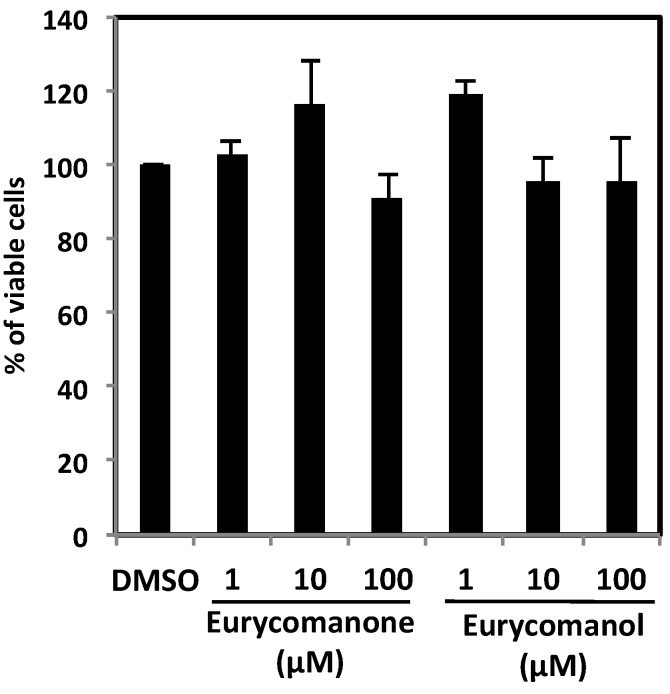
The effect of eurycomanone and eurycomanol on the viability of healthy cells. PBMCs from healthy donors were treated with indicated concentrations for 24 h. DMSO corresponds to solvent-treated control. Each value is a mean ± SD of at least three independent experiments.

To assess differential toxicity and potential side effects triggered by these compounds against circulating healthy cells, the effect of eurycomanone and eurycomanol on peripheral blood mononuclear cells (PBMCs) from healthy donors was investigated. PBMC cell viability was determined by the trypan blue exclusion test after 24 h treatment with various concentrations of either eurycomanone or eurycomanol. PBMC cell viability was above 90% for each concentration and compound tested, indicating that both compounds were not toxic towards healthy cells, but selective against cancer cells (Figure 6). As healthy PBMCs do not proliferate in a physiological setting, we did not stimulate these PBMCs, and so, we did not assess the effect of eurycomanone and eurycomanol on PBMCs artificially triggered to proliferate.

#### 2.1.2. Eurycomanone and Eurycomanol Inhibit TNFα-Induced NF-κB Pathway Activation

The NF-κB pathway is tightly linked to cancer development, progression and resistance against apoptotic cell death. As both compounds decreased proliferation and induced apoptosis, the underlying molecular mechanisms were studied. Jurkat and K562 cell lines were transfected with an expression plasmid coding for luciferase gene under the control of a promoter with an NF-κB response element.

**Figure 7:**
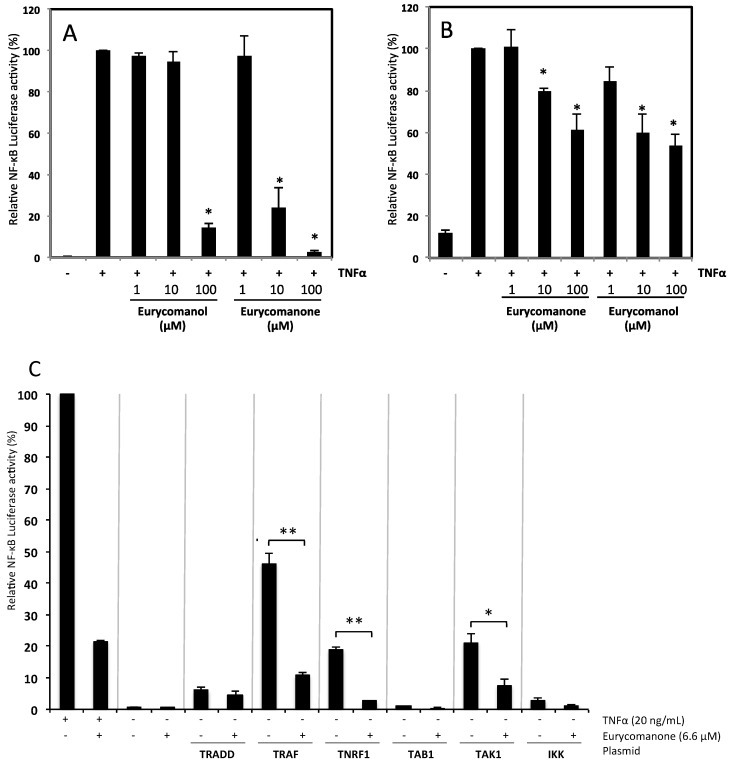
The effect of eurycomanone and eurycomanol on TNFα-induced NF-κB transcriptional potential. K562 (**A**) and Jurkat (**B**) cells were transfected with a luciferase construct under the control of NF-κB. Cells were pre-treated during two hours with compounds then treated, or not, with TNFα (20 ng/mL) for six hours. Each value is the mean ± SD of three independent experiments. * indicates a significant difference compared to the positive control (TNFα alone) analyzed by the *t*-test (* *p* < 0.05). (**C**) Co-transfection of K562 cells with an NF-κB-regulated luciferase reporter construct together with TNFR1-, TRADD-, TRAF2-, TAK1-, TAB1- and IKKβ-expressing plasmids. Each value is the mean ± SD of three independent experiments. The asterisk indicates a significant difference compared to the negative control analyzed by the *t*-test (* *p* < 0.05, ** *p* < 0.01).

For Jurkat cells, the concentration causing 50% inhibition of TNFα-induced NF-κB luciferase activity (IC_50_) was 45 µM for eurycomanone and 100 µM for eurycomanol (Figure 7B). IC_50_ values for K562 cells were 6.6 and 35.6 µM for eurycomanone and eurycomanol, respectively (Figure 7A). Based on these initial results, eurycomanone appears more active than eurycomanol. Moreover, K562 cells are more sensitive to the treatment than Jurkat cells.

To further investigate the cell signaling pathways affected by eurycomanone, we overexpressed essential intermediates implicated in the activation of the NF-κB pathway, including TNFR1, TRADD, TRAF2 and IKK, eventually leading to IκBα phosphorylation. Moreover, formation of TAK1, TRAF2, IKKα and IKKβ complexes is required for IKK activation. Thus, we also transiently co-transfected K562 cells with TAK1-, TAB1- and IKKβ-expressing plasmids along with a NF-κB-regulated luciferase reporter construct. Our results showed that eurycomanone significantly suppressed NF-κB reporter activity in cells co-transfected with TNFR1, TRAF2 or TAK1, but had no inhibitory effect on the activity in cells overexpressing TRADD, IKKβ or TAB1 (Figure 7C). These results suggest TNFR1 and the primary signaling complex as targets of eurycomanone.

#### 2.1.3. Eurycomanone Inhibits TNFα-Dependent Degradation of IκBα and Prevents p50/p65 Nuclear Translocation

Jurkat cells activated NF-κB via a canonical pathway involving the degradation of the IκB inhibitor of the NF-κB transcription factor, allowing its translocation into the nucleus and transactivation of target genes; K562 cells rely on oncokinase Bcr-Abl. According to Baldwin and colleagues. “Expression of Bcr-Abl leads to activation of NF-κB-dependent transcription by causing nuclear translocation of NF-κB as well as by increasing the transactivation function of the RelA/p65 subunit of NF-κB. Importantly, this activation is dependent on the tyrosine kinase activity of Bcr-Abl” [21]. In our hands and in other labs, degradation of the IκB inhibitor is not observable in K562 and accordingly was not investigated here. Nevertheless, as our lab essentially investigates the effects of anticancer compounds on leukemia and lymphoma cell models and as chronic myelogenous leukemia patients show increased resistance against the clinically used Gleevec Bcr-Abl inhibitor, thus requiring novel inhibitors, we use here this essential cellular model compared to Jurkat cells with a canonical activation of NF-κB.

**Figure 8:**
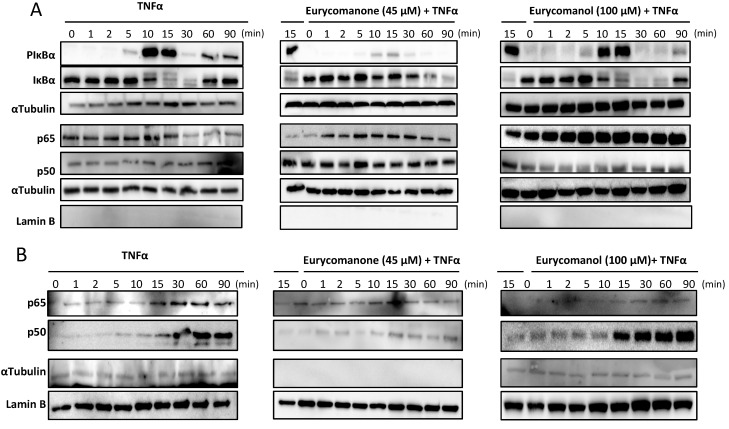
The effect of eurycomanone and eurycomanol on IκBα degradation and on p65 and p50 nuclear translocation. Jurkat cells were pre-treated with eurycomanone (45 μM) and with eurycomanol (100 μM) for two hours followed by activation with TNFα (20 ng/mL) for the indicated time periods. Cytoplasmic (**A**) and nuclear (**B**) extracts were tested for P-IκBα, IκBα, p50 and p65. Protein loading and purity of extracts were verified by lamin B and α-tubulin controls. Data are representative of three independent experiments.

Accordingly, Jurkat cells were treated with eurycomanone and eurycomanol for two hours at 45 and 100 µM, respectively, and then activated with TNFα. Expression levels of IκBα, phosphorylated IκBα, NF-κB subunits p50 and p65, as well as lamin B and α-tubulin loading controls were analyzed by western blots. Eurycomanone inhibited IκBα phosphorylation after 5–15 min in control TNFα-treated cells, whereas eurycomanone did not change the phosphorylation level. Subsequent IκBα degradation observed in control cells after 10–30 min was totally abrogated by eurycomanone treatment, but not by eurycomanol (Figure 8A). Moreover, eurycomanone treatment clearly inhibited translocation of p65 and p50 subunits to the nucleus (Figure 8B), thus contributing to the understanding of the differential inhibition of transactivation, as described above (Figure 7).

#### 2.1.4. Eurycomanone Inhibits MAPK Activation

**Figure 9:**
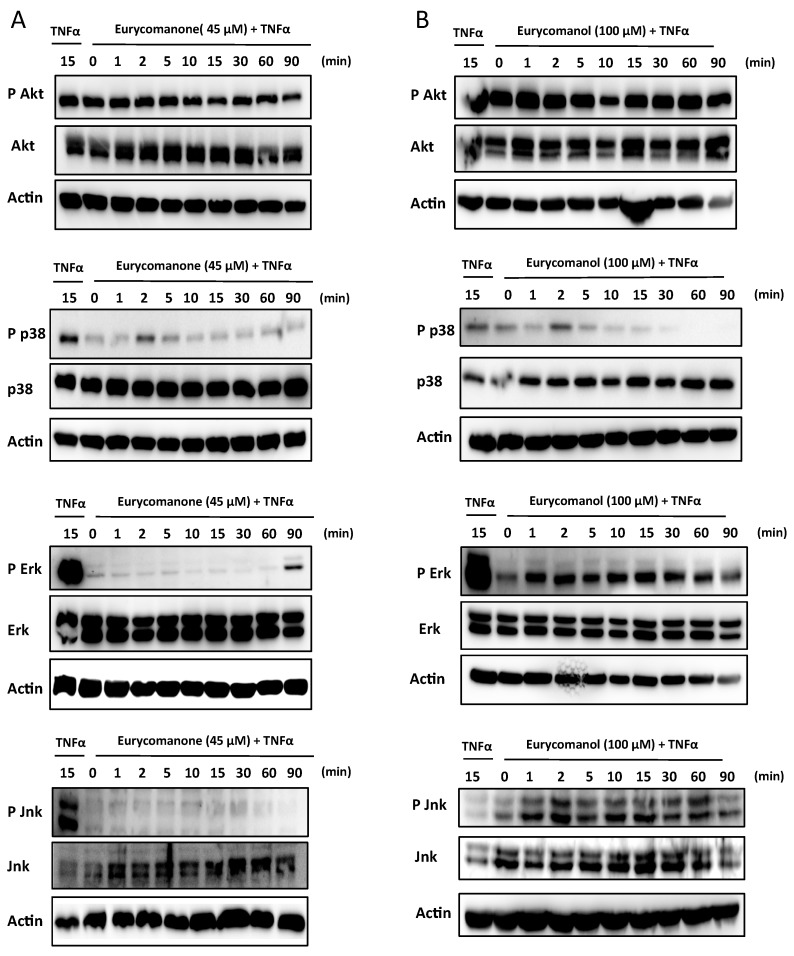
The effect of eurycomanone and eurycomanol on the activation of the MAPK and PI3K/Akt pathways. Jurkat cells were pre-treated with eurycomanone (45 μM) (**A**) and eurycomanol (100 μM) (**B**) for two hours followed by activation with TNFα (20 ng/mL) for the indicated time periods. Data are representative of three independent experiments.

In order to provide further insight into the molecular mechanisms leading to decreased proliferation rates, we hypothesized that mitogen activated protein kinase (MAPK) and phosphoinositide 3-kinase (PI3K)/AKT could be affected by eurycomanone treatment. Our results showed that eurycomanone inhibited both p38 and c-Jun N-terminal kinase (JNK) phosphorylation and delayed phosphorylation of extracellular regulated kinase (ERK), which appeared after 90 minutes compared to the positive control treated by TNFα alone. However, we could not observe any inhibitory effect on PI3K/AKT phosphorylation (Figure 9A). Again, eurycomanol shows a less pronounced effect on kinase phosphorylation in comparison to eurycomanone (Figure 9B).

### 2.2. Discussion

*Eurycoma longifolia* Jack is used in Malaysian traditional medicine and is well known for its beneficial antimalarial effects [2,22]. Moreover, anticancer potential against lung cancer cells was previously demonstrated [23,24]: treatment of lung cancer cells with eurycomanone resulted in decreased prohibitin activity and p53 expression. Both proteins regulate the cell cycle, proliferation and apoptosis. Moreover eurycomanone acts on breast cancer cells by suppressing the expression of breast cancer markers [11]. Here, we investigated the effect of these compounds on the proliferation and survival of leukemia cells, as well as related cell signaling mechanisms.

Interestingly, many compounds of medicinal *Eurycoma longifolia* Jack are already well known for their anti-cancer effects. Induction of tumor promoters by the Epstein-Barr virus is inhibited by 14,15β-dihydroxyklaineanone. Other quassinoids of eurycomanone, such as the 12-acetyl-13,21-dihydroeurycomanone and 14,15β-dihydroxyklaineanone,, showed cytotoxic effects on KB cells; however, only the 14,15β-dihydroxyklaineanone showed activity on leukemic P388 cells. Eurycomalactone, 6α-hydroxyeurycomalactone, 5,6-dehydroeurycomalactone, longilactone, 11-dehydroklaineanone, 14,15β-dihydroxyklaineanone and certain triterpenes, such as niloticin, dihydroniloticin, piscidinol A, bourjotinolone A, 3-episapelin A, melianone and hispidone, also affect KB and P388 cells. Finally, eurylene, 14-deacetyl eurylene and longilene peroxide, three squalene-type triterpenes isolated from roots, are active on P388 and KB cells [22]. Recently, pre-clinical studies in rats have shown that 9-methoxycanthin-6-one, an alkaloid known for its cytotoxic effects, had a very low bioavailability (less than 1% absorption) by oral administration in rats [25]. The main objective of this study was to understand and identify the mechanisms of action of eurycomanone and eurycomanol, two natural compounds from the roots of *E. longifolia*. Both quassinoid structures differ only by a lactone group (eurycomanone) *vs.* a hydroxyl group (eurycomanol) on the functional group in C2.

Our initial findings showed cytotoxic and cytostatic effects of these two compounds on Jurkat and K562 cells, whereas healthy circulating blood cells were not affected. Cell viability is closely linked to cellular survival mechanisms. In addition to playing an important role in inflammation, NF-κB is also involved in cell resistance against apoptosis. Inhibition of this pathway therefore contributes to explaining the effects of the two compounds on cell viability and also their anti-inflammatory effect. Our results showed a dose-dependent decrease of the NF-κB transactivation potential. From these first results, we noted that eurycomanone is more active than eurycomanol. In addition, K562 cells are more sensitive to the treatment than Jurkat.

Considering the presence of the lactone function in NF-κB inhibitors, e.g., salinosporamide A and goniothalamin, the inhibitory activity observed for both compounds was not surprising. It is interesting to note that the α,β-unsaturated ketone group present in eurycomanone is similar to natural products, such as goniothalamin, kavain, curcumin and helenalin, efficiently inhibiting NF-κB. In addition, eurycomanone is a compound from the quassinoid family, exhibiting anti-NF-κB inhibitory potential [26]. Eurycomanone inhibited the NF-κB signaling pathway by inhibiting the phosphorylation of IκBα. This pathway is also inhibited by goniothalamin possessing a similar α,β-unsaturated ketone group as eurycomanone. With regard to eurycomanol, the treatment has no effect on the inhibition of the phosphorylation of IκBα. This difference is possibly again due to the absence of the α,β-unsaturated ketone in eurycomanol compared to eurycomanone and other NF-κB inhibitors.

The effect of both eurycomanone and eurycomanol on the MAPK and PI3K/Akt signaling pathways involved in cell survival and proliferation were also evaluated. Our results showed an inhibition of p38 and JNK phosphorylation with decreased and delayed ERK phosphorylation after eurycomanone treatment. There is no effect on the phosphorylation of AKT. Treatment with eurycomanol remained without effect on these pathways. Nevertheless, inhibition of the MAPK pathway by eurycomanone explained the effect of both compounds on the proliferation and cell viability observed earlier.

## 3. Experimental Section

### 3.1. Plant Material

Roots were collected from Tapah, Perak, and were authenticated by Emeritus Professor Dato’ Dr. Abdul Latif Mohamed, Universiti Kebangsaan Malaysia and deposited in UiTM with a voucher specimen code (FP/UiTM/EL/3/10). The powdered roots (2 kg) of *E. longifolia* were macerated with 70% aqueous methanol at 40 °C for 8 h. Aqueous methanolic extract was filtered and dried under reduced pressure at 40 °C. The extraction process was repeated five times, and the combined aqueous methanolic extract (52 g) was sequentially partitioned with *n*-hexane, chloroform and *n*-butanol.

### 3.2. Isolation of Pure Compounds

The *n*-butanol (5.0 g) fraction was subjected to open silica gel (No. 9385, Merck, Darmstadt, Germany) column chromatography with methanol:chloroform mixtures in ratios of 9:1, 7:3 and 1:1. Five fractions with similar R_f_ values on TLC were pooled together. Fraction 2 was further purified by a Waters Autopurification™ System connected to an X-Bridge C18, 5 μm, 150 × 19 mm (Waters, Milford, MA, USA) monitored at 235 nm, affording eurycomanone (32 mg). The mobile phase used was a mixture of acetonitrile:water (3:7) at 3 mL/min. Repeated recrystallization of Fraction 3 with methanol afforded eurycomanol (16 mg). The structures and purity (>98%) of the isolated compounds were confirmed by comparison with reported optical rotation, NMR, MS, UV and IR spectroscopic data [27,28].

### 3.3. Cell Culture

K562 (human chronic myelogenous leukemia) and Jurkat (T-cell leukemia) cells (Deutsche Sammlung von Mikroorganismen und Zellkulturen GmbH, DSMZ) were cultured in RPMI 1640 medium (Lonza, Verviers, Belgium) supplemented with 10% (v/v) fetal calf serum (Lonza, Verviers, Belgium) and 1% (v/v) antimycotic (Bio-Whittaker, Verviers, Belgium) at 37 °C with 5% CO_2_, in a humidified atmosphere. Cells were harvested every 3 days. Exponentially growing cells were treated with compounds as indicated for respective experiments.

Healthy blood samples were kindly donated as buffy coats by the Red Cross (Luxembourg, Luxembourg). Blood was diluted in RPMI 1640 and applied onto a Ficoll layer (GE Healthcare, Diegem, Belgium) and further centrifuged (400× g, 20 min) to isolate mononuclear cells. Isolated PBMCs were cultured at 37 °C with 5% CO_2_ for 24 h before they were subjected to drug treatments.

### 3.4. Cell Viability Assessment

K562 and Jurkat cells were incubated with various concentrations of eurycomanone and eurycomanol during 8 h, 24 h, 48 h and 72 h. Cell viability was assessed by the trypan blue exclusion test (Lonza, Verviers, Belgium). PBMCs viability was assessed 24 h after treatment with both compounds by the CellTiter-Glo^®^ Luminescent Cell Viability Assay kit (Promega, Leiden, The Netherlands), according to the manufacturer’s instructions. Data were normalized to the control and reported as the percentage of viable cells.

### 3.5. Proliferation Assay

The effect of eurycomanone and eurycomanol on K562 and Jurkat cell proliferation was monitored in real-time using the IncuCyte^TM^ Live-Cell Imaging System (Essen BioScience, Hertfordshire, United Kingdom). After seeding in a 24-well plate coated with poly-d-lysine hydrobromide, K562 and Jurkat cells were treated with different concentrations of eurycomanone and eurycomanol. The IncuCyte^TM^ microscope permits the acquisition of automated phase contrast images. Individual images are processed by an imbedded contrast-based confluence algorithm, which compute monolayer confluence for each image and at each time point. Multiple images are collected per well and averaged to provide a representative statistical measure of confluence.

### 3.6. Analysis of Nuclear Fragmentation

K562 and Jurkat cells were treated with different concentrations of eurycomanone and eurycomanol during 8, 24, 48 and 72 h. Percentages of apoptotic cells, quantified as the fraction of cells presenting fragmented nuclei, were assessed by fluorescence microscopy (Leica DM IRB microscope, Lecuit, Luxembourg) upon staining with the DNA-specific dye Hoechst 33342 (Sigma–Aldrich, Bornem, Belgium), as previously described [29]. The fraction of cells with nuclear apoptotic morphology was counted (at least 300 cells in at least three independent fields).

### 3.7. Cell Cycle Analysis

The cell cycle was analyzed according to standard procedures. Briefly, eurycomanone- and eurycomanol-treated K562 and Jurkat cells were fixed with an ethanolic solution in water (70% v/v), and DNA was stained with propidium iodide (1 μg/mL; Becton Dickinson Biosciences, Erembodegem, Belgium) and RNAse A (100 μg/mL; Roche, Luxembourg) in PBS. Events were recorded statistically (10,000 events/sample) using the CellQuest software associated with FACSCalibur (BD Biosciences). Data were further analyzed by using FlowJo software.

### 3.8. Transient Transfection and Luciferase Reporter Gene Assay

K562 and Jurkat cells were transiently transfected, as described previously [12]. For each electroporation, 5 μg of a luciferase reporter gene construct containing 5 repeats of a consensus NF-κB site (Stratagene, Genomics Agilent, Diegem, Belgium) and 5 μg of a *Renilla* luciferase plasmid (Promega, Leiden, The Netherlands) were added to the cells. For co-transfection assays, 5 μg of expression plasmids coding for proteins of the NF-κB pathway (receptor of TNFα-1 (TNFR-1), TNFR-associated factor-2 (TRAF2), TNFR-1 associated death domain (TRADD), inhibitor of κB kinase beta (IKKβ), tumor growth factor activated kinase 1 (TAK1) and TAK1-binding protein 1 (TAB1)) were added to the electroporation mixture. Twenty four hours after transfection, cells were re-suspended in fresh growth medium (RPMI 1640, 0.1% FCS, 1% AB) to reach a final concentration of 1 × 10^6^ cells/mL and subjected to a 2-h pre-treatment with eurycomanone and eurycomanol followed by 6 h of NF-κB-activation by 20 ng/mL of TNFα. For co-transfection experiments, TNFα was used as a control. To assay firefly luciferase activity, 75 µL of Dual-Glo^TM^ Luciferase Reagent (Promega, Leiden, Netherlands) were added to 75 µL of the cellular suspension for 10 min incubation at 22 °C. Seventy five microliters of Dual-Glo^TM^ Stop&Glo^®^ Reagent (Promega, Leiden, The Netherlands) were then added for 10 min at 22 °C for measurement of *Renilla* activity using the microplate luminometer (Orion, Berthold, ND, USA). Results are expressed as the ratio of arbitrary units of firefly luciferase activity to *Renilla* luciferase activity.

### 3.9. Extraction of Cellular Proteins

At the end of treatment with eurycomanone and eurycomanol with or without activation with TNFα, K562 and Jurkat cells were lysed, and nuclear and cytoplasmic fractions were extracted [12] Briefly, cell pellets (10^7^ cells per sample) were suspended in ice-cold hypotonic lysis buffer containing a protease inhibitor cocktail (Roche, Luxembourg, Luxembourg) and incubated on ice for 15 min. Protein content was determined for each sample using the Bradford assay (Bio-Rad protein Assay, Bio-Rad, Nazareth, Belgium).

### 3.10. Western Blot Analysis

Nuclear and cytoplasmic proteins fractions were subjected to sodium dodecyl sulfate polyacrylamide gel electrophoresis (SDS-PAGE, 10%) and transferred onto nitrocellulose. Membranes were pre-hybridized with 5% non-fat milk in phosphate buffered saline (PBS)-Tween overnight. Lamin B and α-tubulin were respectively used as loading controls for cytoplasmic extracts and nuclear extracts, Hybridizations with primary antibodies were directed against pIκBα (Ser32), ERK, pERK (Thr202/Tyr204), JNK, pJNK (Thr183/Tyr185), p38, pp38 (Thr180/Tyr182), Akt, pAkt (Ser473) (Cell Signaling, Bioké, Leiden, The Netherlands), IκBα (Santa Cruz, Tebu-Bio, Boechout, Belgium), p50 and p65 (Santa Cruz), α-tubulin (Calbiochem, VWR, Leuven, Belgium) or lamin B (Santa Cruz). All antibodies were diluted in a PBS-Tween solution containing 5% bovine serum albumin (BSA) or 5% milk, according to the providers’ protocols. After incubation with primary antibodies, membranes were washed with PBS-Tween followed by an incubation of 1 h at room temperature with the corresponding secondary (HRP-conjugated) antibodies. Specific immunoreactive proteins were visualized by autoradiography using the ECL Plus Western Blotting Detection System Kit^®^ (GE Healthcare, Roosendaal, The Netherlands).

### 3.11. Statistical Analysis

Results from at least three independent experiments were statistically analyzed by a Student’s paired *t*-test using GraphPad Prism version 6.00 (GraphPad Software, La Jolla, CA, USA). Results from the IncuCyte video microscopy-based approach were also statistically analyzed with a two-way ANOVA. *p*-values below 0.05 (*), 0.01 (**) or 0.001 (***) were considered as significant.

## 4. Conclusions

Eurycomanone is a molecule with a very promising bioactivity, as it prevents the induction of NF-κB and MAPK by TNFα, without strongly affecting the viability of healthy cells. In addition, eurycomanol does not inhibit either NF-κB or MAPK. However, it has a limited activity on cell proliferation and the induction of apoptosis most likely by targeting different signaling pathways, which remain to be investigated by future studies. In that sense, potential inhibition of selected metabolic pathways by eurycomanone is a highly interesting perspective deriving from our studies here, and the effect of the selected quassinoids on energy-related mechanisms will be assessed by future investigations.

## Supplementary Files

Supplementary File 1
